# Early Introduction of Solid Feeds: Ingestion Level Matters More Than Prebiotic Supplementation for Shaping Gut Microbiota

**DOI:** 10.3389/fvets.2020.00261

**Published:** 2020-05-15

**Authors:** Charlotte Paës, Thierry Gidenne, Karine Bébin, Joël Duperray, Charly Gohier, Emeline Guené-Grand, Gwénaël Rebours, Olivier Bouchez, Céline Barilly, Patrick Aymard, Sylvie Combes

**Affiliations:** ^1^GenPhySE, Université de Toulouse, INRAE, ENVT, Castanet-Tolosan, France; ^2^CCPA, Janzé, France; ^3^EVIALIS, Lieu dit Talhouët, Saint-Nolff, France; ^4^MiXscience, Bruz, France; ^5^Wisium, Chierry, France; ^6^TECHNA, Couëron, France; ^7^GeT-PlaGe, Genotoul, INRAE, Castanet-Tolosan, France

**Keywords:** early life, rabbit, microbiome, weaning, gel starter diet, oligosaccharide, FOS, MOS

## Abstract

Early introduction of a nutritional substrate is a promising biomimetic strategy for controlling the implantation of the microbiota and preserving the health of young animals. In this study, we provided experimental solid substrate in a gel form to stimulate suckling rabbits' intake and to investigate its effects on microbiota implantation and colonization. All the rabbits had access to solid feed outside the nest as of 15 days of age. Except for the control group, rabbits were offered starter feed gels inside the nests from 3 to 18 days of age. These gels were either free of additives (AF_GEL) or contained 4% of fructo-oligosaccharides (FOS_GEL) or 4% of mannan-oligosaccharides and β-glucans mixtures (MOS_GEL). The cecal content of 160 rabbits was sampled at 18, 29, 38, and 57 days of age and analyzed using 16S rRNA gene sequencing. Pups consumed an average of 3.95 ± 1.07 g of starter feed gel with a higher intake when it was supplemented with fructo-oligosaccharides (+1.2 g; *P* < 0.05). Starter feed gel consumption increased the ensuing intake of pellets (+17 g from 15 to 21 days; *P* < 0.05). Alpha-diversity indexes were similar between groups and prebiotic supplementation did not induce a clear shift in microbiota pattern. Conversely, when considering rabbits that consumed more starter feed, the highest proportions of bacteria with plant-degrading abilities, such as species from the Lachnospiraceae and Ruminococcaceae families, were observed at 18 days of age. However, fermentative activities were not affected by starter feed intake at 29, 38, and 57 days of age. By providing comprehensive results on the regulation of microbial community structure at the onset of solid feed intake, this research paves the way for further studies on digestive ecosystem maturation.

## Introduction

Young mammals are prone to enteritis around weaning (e.g., pigs, ruminants, rabbits, etc.), which represents a stressful period due to the end of milk consumption and the separation from the mother. Although the etiology of these digestive disorders is not yet clear, gut microbiota may play a pivotal role. Indeed, disruption of the gut microbiota homeostasis, characterized by the greater abundance of opportunistic and pathogenic species and the decreased abundance of fibrolytic and butyrate-producing bacteria, appears to predispose the young animals to enteric infections (1–3). On the contrary, when an equilibrium between mammalian host and gut microbiota is established, the microbiota will contribute to host defense in different ways (4). In particular, the gut microbiota prevents the establishment of detrimental bacteria through competition for shared nutrients and niches (5). In addition to this “barrier” function, numerous studies have underlined the action of the gastrointestinal microbiota on the immune system (6). Early-life exposure to the gut microbiota is determinant, in particular, for the establishment of a normal immune function (7, 8).

Longitudinal studies in young mammals underlined highly variable gut taxonomies between neonatal animals, followed by the establishment of “climax” communities with greater uniformity in late post-weaning states [humans: (9); rabbits: (10); pigs: (11); calves: (12)]. As a consequence of this immaturity, the early post-natal stage represents a period of permissiveness that has been described as a “window of opportunity” for microbiota and immune system engineering (13). Gut microbiota manipulation in early life can be performed with different tools, including dietary interventions. Indeed, numerous studies have shown that the introduction of solid feed strongly affects the gut microbiota (14–16) since dietary nutrients provide substrates for microbial growth.

The rabbit pup is a good model to study early dietary intervention incidence on gut microbiota installation. Unlike other altricial species, wild rabbit pups are able to ingest solid substrates shortly after birth in the burrow (17). Under controlled breeding conditions, it was demonstrated that pups exhibit a coprophagous behavior within the first week of life (18, 19) and consume the feed provided in the nest as of 8 days of age (20, 21). The ability of the suckling rabbit to consume feed at an early stage can therefore be used as a biomimetic strategy to study the intestinal colonization of commensal bacterial communities.

Little is known about the effects of the quality of solid feed ingested in early life on gut microbiota, but the use of prebiotics to target bacterial groups of interest appears promising. Prebiotics contribute to digestive health preservation by functioning as anti-adhesives, preventing pathogen implantation, stimulating immune maturation and gut barrier function, and serving as fermentable substrates for gut bacteria. The use of mannan-oligosaccharides (MOS) in pre- and post-weaning rabbit diets effectively promotes cecal fermentation and gut barriers (22–24). Moreover, prebiotics are present in mammal's milk (25–27) and are essential to support the development of the commensal microbiota in infants. In humans, fructo-oligosaccharides (FOS) are routinely added to infant formulas to mimic the beneficial effects of milk oligosaccharides on the commensal microbiota (28). Consequently, maintaining prebiotic supplementation at the milk/solid diet transition by providing non-digestible carbohydrates in the starter diet might ensure the installation of a proper ecological succession of bacterial species.

We hypothesized in this study that an early solid dietary intervention would drive microbiota establishment toward the installation of beneficial microorganisms and that prebiotic supplementations would potentiate those effects. In agreement with our previous findings (29), a hydrated starter diet was given to suckling rabbits to stimulate early solid ingestion. The effects of solid feed supplemented or not with prebiotics (FOS or MOS) from 3 to 18 days of age were assessed in terms of animal development, microbiota colonization, and fermentative activity up to 57 days of age. To our knowledge, this study is the first to investigate the effects of post-natal solid feed supplementation on rabbit microbiota structure.

## Materials and Methods

### Ethics Statement

This study was carried out at the PECTOUL Experimental Unit (INRAE, Castanet-Tolosan, France). Animals were raised and handled according to the European Union's recommendations concerning the protection of animals used for scientific purposes (2010/63/EU) and in agreement with French legislation (NOR: AGRG1238753A 2013). The experiment received the approval of the local ethics committee (SSA_2019_001).

### Animal Management

#### Animal Handling and Housing

Commercial rabbits raised for meat production were used in the experiment (crossbreed of the maternal line Hyplus PS19 and the paternal line Hyplus PS59; Hypharm, France). Before weaning, the does were housed individually with their pups in wire cages (width: 62 cm; length: 69 cm; height: 62 cm) equipped with nests for the pups (width: 25 cm; length: 38 cm; height: 20 cm), as previously reported (29). Two days after parturition (d2), nest quality was assessed on a 3-point scale, i.e., a nest of good quality corresponded to a nest that was fully covered by doe fur. After nest quality assessment, the litter size was standardized to ten pups per doe by cross-fostering or culling. From 3 to 21 days, nursing was controlled and pups had access to milk once a day. All the rabbits received commercial feed pellets *ad libitum* from 15 to 35 days in a feeder designed for young rabbits (30) that the doe could not reach. At weaning (d35), pups were assigned to collective cages of 5 rabbits and mixing rabbits from different litters was prevented. Until d64, they were fed the same commercial post-weaning diet restricted at 79% of the *ad libitum* intake (31). No antibiotics were provided to pups and their mothers throughout the experiment. Chemical composition analysis was performed on the commercial diets using ISO methods (DM and ash for dry feed: ISO 6496:1999; nitrogen content: ISO 16634-1:2008; crude fat content: ISO 6492:1999; gross energy: ISO 9831:1998) and the procedures described by the European Group on Rabbit Nutrition [(32); Table 1]. The litter weight after suckling was recorded at 3, 14, 21, and 28 days of age. Rabbits were individually weighed at weaning (d35), 50, 64, and 71 days of age.

**Table 1:** Ingredients and chemical composition of the commercial diets provided.

	**Before weaning**	**After weaning**
**Ingredients (g/kg as-fed basis)**		
Wheat bran	301	251
Wheat		84
Sunflower meal (high protein)	236	101
Sunflower meal (low protein)		77
Sunflower husk	89	100
Barley	174	
Sugar beet pulp	95	187
Sugar beet molasses	50	50
Alfalfa		80
Rapeseed meal	30	30
Rapeseed oil	2	3
Calcium carbonate	12	19
Salt	6	7
Fortibut +		5
L-Lysine monochloride	1	1
Mineral and vitamin premix	5	5
**Chemical composition (g/kg as-fed basis)**		
Ash	64	87
Crude protein	176	154
Neutral detergent fiber (NDF)	319	375
Acid detergent fiber (ADF)	165	205
Acid detergent lignin (ADL)	51	64
Hemicellulose (NDF-ADF)	154	171
Cellulose (ADF-ADL)	114	140
Starch	131	78
Fat content	25	19
Gross energy (kcal/kg)	3,917	3,803

#### Experimental Groups

**Figure 1:**
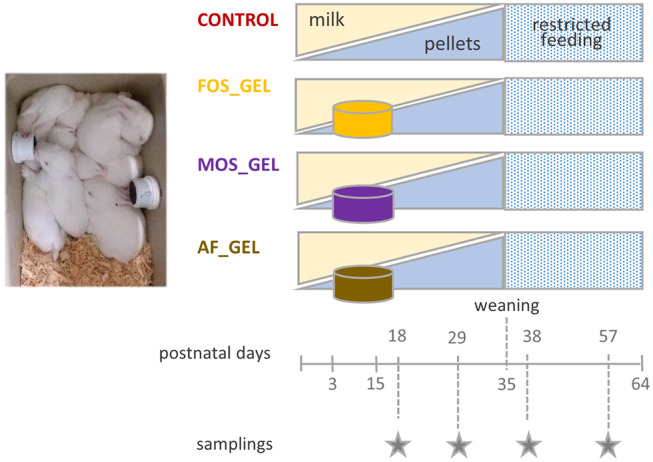
Experimental design: three groups of 11 rabbit litters were provided with a starter feed in a gel form from 3 to 18 days of age in addition to doe milk. The additive composition of the gels varied according to treatments (CONTROL, no starter feed gel provided; FOS, fructo-oligosaccharides; MOS, a mixture of mannan-oligosaccharides and β-glucans; AF, no additive in the starter feed gel).

A total of 44 litters were equally distributed between four experimental groups at 3 days of age by stratified randomization based on the does' parity (multiparous does, an average of five parities), litter weight at standardization (72 ± 7 g) and the allocation in the farm (*n* = 3 rooms). In the CONTROL group, rabbits had access to solid feed as of day 15 with commercial pellets. An additional starter feed was offered to the three other groups in a hydrated gel form from 3 to 18 days in two plastic cups (volume: 30 mL; Ø = 40 mm; height: 32 mm; GOSSELIN®, Le Mans, France) that were vertically clipped to each side of the nest (Figure 1). The gels were removed before suckling to prevent the doe from eating them. The starter feed gels were renewed every day and their consumption was measured as of 7 days of age. To process the gels, the commercial pellets provided during the pre-weaning period were first mashed (particle sizes smaller than 2 mm). An attractive flavoring additive was added to the mash (vanilla flavor at 0.06%, supplied by Phodé, Terssac, France). For diets including prebiotics, the corresponding additive (powder form) was also mixed with the mash at this stage. Dry products were then thoroughly mixed with hot water (80–90°C) and agar to shape the gels (mash-to-water ratio of 1:4 with 0.6% of agar). Litters that received starter feed gels without an additive belonged to the AF_GEL (Additive-Free Gel) group. The prebiotics used were either fructo-oligosaccharides (FOS_GEL group) or a mixture of mannan-oligosaccharides and β-glucans (MOS_GEL group). Short chain fructo-oligosaccharides were provided as Profeed® (Tereos, Lille, France). This product is obtained from beet sugar through a bio-enzymatic process. It is characterized by a degree of polymerization between 3 and 5 and is composed of three glucose-fructose chains, resulting in a final concentration of 95% of FOS. Mannan-oligosaccharides and β-glucans combinations such as AGRIMOS® were provided by Lallemand Animal Nutrition (Blagnac, France). This additive is obtained by the autolysis of yeast cell walls of *Saccharomyces cerevisiae*, leading to a final concentration of 45% of MOS. Manufacturers' recommendations for inclusion levels for post-weaned rabbits are typically close to 2 kg per ton of feed (0.2%). Considering the specificity of our experimental design, the additives constituted 0.9% of the gels, the equivalent of 4% of dry matter (DM). The DM content of starter feed gels obtained by freeze-drying for 48 h was similar between gel types (25.6–26.3%). Given the high moisture content of starter feed gels, water losses due to evaporation were evaluated daily for each type of gel. Those autogenic changes in gel mass were taken into account with a correction factor based on the quantity of gel supplied. Feed intake before weaning was measured at the litter level and expressed per rabbit. For feed consumption after 18 days of age, the number of pups was adjusted assuming that dead animals did not consume feed 2 days before their death.

### Measurement of Digestive Tract Development and Cecal Content Sampling

At days 18, 29, 38, and 57, 10 pups per group (one pup per litter) were randomly selected, weighed and killed by electronarcosis and exsanguination (*n* = 160 pups in total). Blood samples were collected at exsanguination in EDTA tubes immediately stored on ice. After centrifugation (800 g for 10 min at 4°C), the plasmas were stored at −20°C until further analysis. The cecum was isolated and weighed before collection of the digesta in sterile tubes (storage at −80°C). At days 29, 38, and 57, cecal pH was measured by introducing a glass electrode at the ileocecal junction (VWR Collection SP225; Radnor, PA, USA) and fresh cecal contents were in sufficient quantities to be sampled for the following analyses: DM (2 g), volatile fatty acids (VFA) (1 g diluted in 2 mL of H_2_SO_4_ at 2% w/v), and ammonia (1 g diluted in 3 mL of H_2_SO_4_ at 2% w/v). The cecum was then emptied and weighed with an OHAUS scale (Parsippany, NJ, USA). Finally, the stomach, small intestine and colon were isolated and weighed.

### Evaluation of the Fermentative Activity of the Cecal Microbiota: Determination of the Concentration of Ammonia and Volatile Fatty Acids

Ammonia (NH_3_) and VFA were analyzed after centrifugation and dilution at 1:10. Ammonia concentrations were quantified using a colorimetric method with a continuous flow analyzer (SAN++; Skalar, Norcross, GA, USA), as previously described (33). Volatile fatty acids were determined by gas chromatography (CPG HP 7890A; Agilent, Santa Clara, CA, USA) following a method previously reported (34). Results were expressed according to the liquid phase of cecal content after determination of cecal sample DM by drying at 103°C for 24 h.

### ELISA Measurements of IgG and IgA

Total plasma IgG or cecal content IgA levels were determined in duplicates by sandwich ELISA in 96-well plates coated with specific polyclonal goat anti-rabbit IgG or IgA antibody (Bethyl Laboratories, Montgomery, Texas, USA) with further plate reading at 450 nm as previously described (35). IgG were quantified by using a reference IgG serum (Bethyl Laboratories). Regarding IgA, 10 samples were pooled to build a reference sample for the standard curve construction.

### DNA Extraction and PCR Amplification of Bacterial 16S Ribosomal Genes for Illumina Sequencing

Total genomic DNA was extracted from 50 mg of cecal digesta using the Quick-DNA Fecal/Soil Microbe 96 Kit (ZymoResearch, Irvine, CA, USA) according to the manufacturer's instructions after mechanical lyses at 30 Hz for 15 min (TisueLyzer II, Qiagen, Hilden, Germany). The 16S rRNA V3-V4 region was amplified by PCR and paired reads were sequenced by MiSeq Illumina Sequencing at the Genomic and Transcriptomic Platform (INRAE, Toulouse, France), as previously described (36).

### Sequence Analysis

The Galaxy-supported FROGS pipeline (37) was used to process the 7,503,813 16S ribosomal DNA amplicon sequences obtained. Amplicons without any ambiguous base, with a length between 350 and 500 nucleotides and matching V3 and V4 proximal PCR primer sequences, were kept for clustering. Reads were clustered into OTUs (operational taxonomic units) using the iterative growth process SWARM (aggregation distance = 3) (38). Chimera were detected using VSEARCH (39) and then discarded (15.3% of the total sequences corresponded to 41.8% of the total OTUs determined). Remaining OTUs were filtered to keep OTUs present in at least three samples, representing more than 0.005% of all of the sequences.

**Figure 2:**
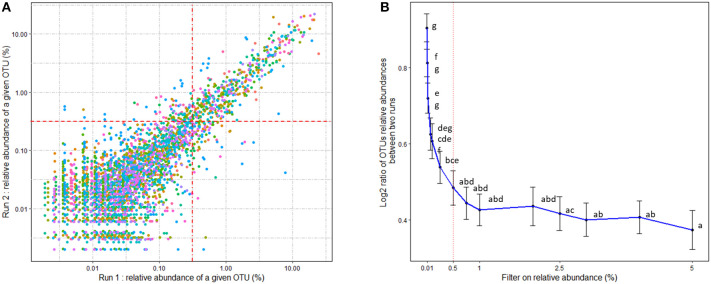
**(A)** Comparison of OTU relative abundances obtained from 15 rabbit cecal samples extracted and sequenced two times. Technical procedures and bioinformatic treatments were similar for replications. Colors stand for the different rabbit samples. Axes are represented on a log10 scale. **(B)** Evaluation of a threshold on OTU relative abundances for quantitative repeatability. Binary log ratio of the relative abundances of each OTU on the two extractions and sequencing analyses for a given sample were calculated as a function of a selected filter on the relative abundance. Means followed by a common letter are not significantly different according to the Tukey HSD test at the 0.05 significance level. The minimum threshold filter of 0.5% was selected.

The mean number of reads per sample was 30,025 (min: 14,110; max: 61,030). OTU taxonomic affiliation was performed using the BLAST algorithm against the SILVA SSU Ref NR 132 database with a pintail quality of 80 (40). Within-community diversity metrics (α-diversity), including Shannon and Inverse Simpson, were calculated after rarefaction of the OTU table at 14,110 sequences (41). In order to evaluate the repeatability of the relative abundances of the OTUs, 16S rDNA sequences from 15 cecal samples extracted, sequenced and processed twice according to the same procedure were compared. As previously observed (42), the quantification of OTUs with low abundances is poorly repeatable (Figure 2A). We chose the threshold of 0.5% of relative abundances for quantitative repeatability, according to Figure 2B. With such a filter, OTU relative abundance differed by an average of 1.4-fold between two DNA extractions and 16S sequencing analyses of the same sample. As a consequence, the OTUs with average relative abundances below 0.5% within each age^*^treatment group were removed before further statistical analysis.

### Statistical Analysis

All statistical analyses were performed using R software (version 3.5.1). Differences were considered to be significant when *P* ≤ 0.05. The Partial Least Squares Discriminant Analysis (PLS-DA) multivariate regression model was used to identify the OTUs that allow discrimination of the response variable using mixOmics package (43). This model uses OTU counts after Total Sum Scaling normalization and Centered Log Ratio Transformation as input to predict the response variable (either experimental group or early feed intake level). The effects of the groups on the discriminant OTUs identified with PLS-DA were analyzed with multiple regression models by controlling the false discovery rate after fourth root transformation applied on relative abundances. Taxonomic assignments to family and genus level obtained by BLAST were considered with a cutoff of 80% for coverage and 80 and 97% for identity, respectively. Taxonomic relative abundances at phylum, family and genus levels, diversity indexes, cecal and blood parameters, longitudinal intakes, and performance data were analyzed using the linear mixed procedure of the nlme package. The mixed model included age, group and their interaction as fixed effects, litter as a random effect, and a correction for age heteroscedasticity was applied when necessary (Supplementary Table S1). *Post-hoc* comparisons were made with the emmeans R package. The total intake in the nest (from 7 to 17 days) was analyzed using nest quality and experimental group as categorical factors, and litter weight at equalization as the quantitative covariable. Mortality data were analyzed using adjusted chi-squares according to the Donner and Banting procedure (44) so that litter cluster was accounted for Reed (45) and Princée (46).

## Results

### Effects of Starter Feed Gels and Prebiotic Supplementation on Solid Feed Ingestion by Suckling Rabbits

**Figure 3:**
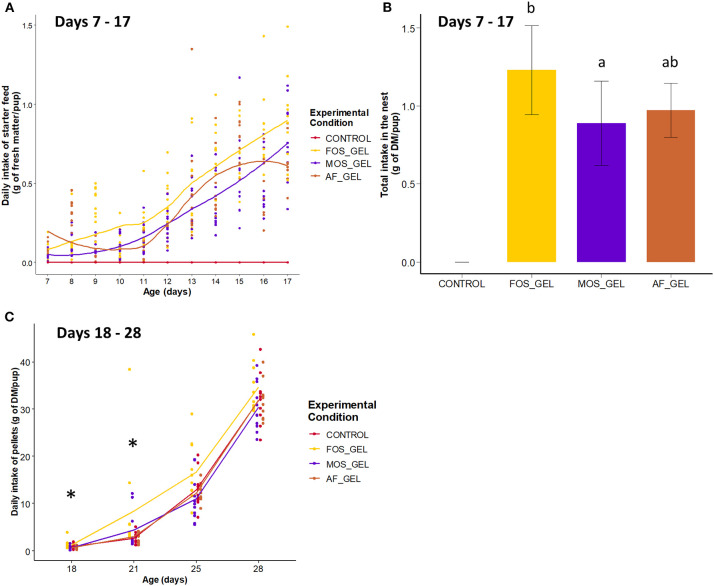
Ingestion of starter feed gels during early life according to treatment and long-term effects of early dietary interventions on subsequent pellet intake before weaning in rabbits. **(A)** Kinetics of starter feed gel ingestion from 7 to 17 days of age in rabbit pups in the nest, where each point stands for one litter's intake. **(B)** Total starter feed gel intake in the nest from 7 to 17 days of age, means with different letters differ at *P* < 0.05. **(C)** Daily Intake of pellets from 18 to 28 days of age in young rabbits. Significant differences between experimental groups found within ages are represented with an asterisk. Error bars represent standard deviation data. CONTROL, no starter feed gel provided; FOS_GEL, fructo-oligosaccharides; MOS_GEL, a mixture of mannan-oligosaccharides and β-glucans; AF_GEL, no additive in the starter feed gel.

The first step of our study was to determine if starter feed gels provided from 3 to 18 days of age promoted early-life solid ingestion in suckling rabbits and if these effects were modulated by prebiotic supplementation (FOS or MOS). Rabbit pups began to ingest the starter feed gels provided in the nest as early as 7 days of age, with high variability between litters (Figure 3A). Daily ingestion was significantly affected by the type of gels provided (*P* = 0.014). Total gel intake in the nest amounted to 3.95 ± 1.07 g of fresh matter and 1.03 ± 0.27 g of DM per pup on average. Total starter feed gel intake in the nest was significantly higher for the FOS_GEL group compared to the MOS_GEL group (+1.3 g of fresh matter; *P* = 0.011) and tended to be higher for the FOS_GEL group than for the AF_GEL group (+1.0 g of fresh matter; *P* = 0.057; Figure 3B). Considering the initial prebiotic concentration of the additives used and their rate of incorporation into the gel, pups from the FOS_GEL group ingested a total of 47 ± 11 mg of FOS/rabbit, whereas those in the MOS_GEL group ingested 16 ± 4 mg of MOS/rabbit. The weight of the litter after standardization (d3, average rabbit weight of 98 ± 10 g) positively affected the total starter feed gel intake (*P* = 0.028; Pearson correlation = 0.41), while the nest quality score did not affect early feed ingestion. All the rabbits had access to similar pellets as of 15 days of age but their intake levels varied according to dietary intervention in early life (Supplementary Table S2). Pre-weaning pellet intake was significantly higher in the FOS_GEL group than in the others, with 17 additional grams of dry matter consumed from 15 to 21 days (*P* < 0.05; Figure 3C). Altogether, our results show that starter feed gels promoted pre-weaning solid feed ingestion in suckling rabbits and that these effects were amplified when the gels were supplemented with the FOS prebiotic.

### Effects of Starter Feed Gels and Prebiotic Supplementation on the Growth and Health of Young Rabbits

**Figure 4:**
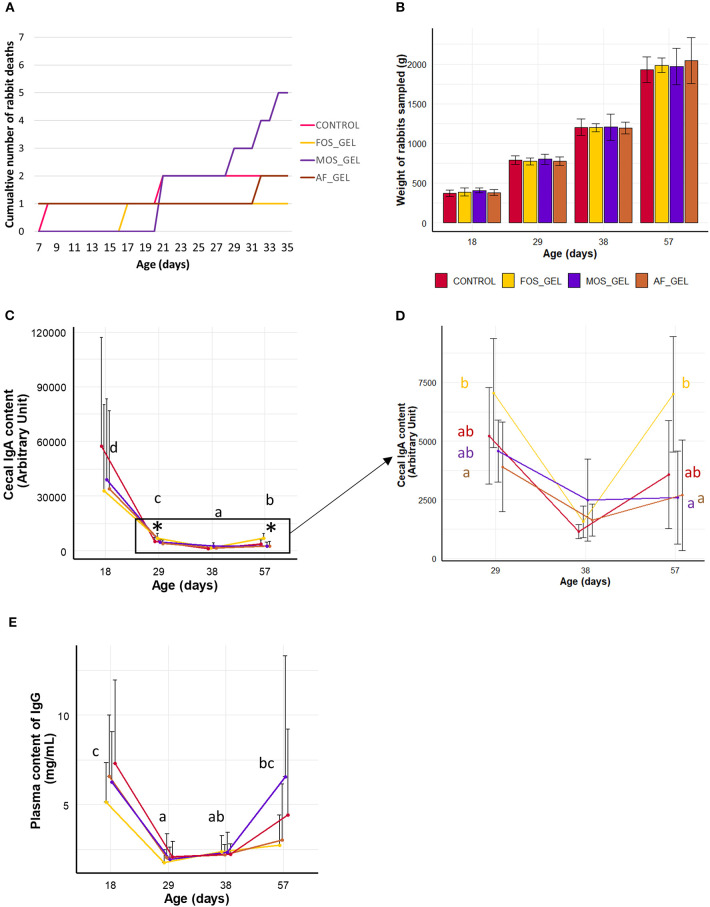
Health parameters and growth of the rabbits according to early dietary interventions in the nest in the form of a starter gel feed. **(A)** Mortality from 7 to 35 days of age. **(B)** Rabbit weight from 18 to 57 days. **(C,D)** Cecal IgA and **(E)** plasma IgG levels in rabbits. The animals sampled for immunoglobulin analyses were healthy and had comparable live weights between groups **(B)**. Significant differences between experimental groups found within ages are represented with an asterisk. Black letters are meant to indicate age effects. Error bars represent standard deviation data. The additive composition of the gels varied according to treatments (CONTROL, no starter feed gel provided; FOS_GEL, fructo-oligosaccharides; MOS_GEL, a mixture of mannan-oligosaccharides and β-glucans; AF_GEL, no additive in the starter feed gel).

In the next step, we analyzed the consequences of the stimulation of solids' ingestion induced by starter feed gels and prebiotics on the growth and health of young rabbits. Thirteen rabbits were found dead shortly after equalization and before rabbit pups began to eat starter gel feed (3–6 day). After this period, mortality was 2.8% from 7 to 35 days and 0.6% after weaning. Survival between 7 and 35 days was numerically lower in the litters from the MOS_gel group (Figure 4A) but this was not significant (χ^2^ = 2.9, *df* = 3, *P* = 0.403). Relative to the CONTROL group, the odds ratios calculated in the FOS_GEL, MOS_GEL, and AF_GEL groups were 0.5 ($\frac{1/89}{2/88}$), 2.7 ($\frac{5/82}{2/88}$), and 1 $\left(\frac{2/88}{2/88}\right)$ respectively. Animal weight before and after weaning and average daily weight gain were not affected by experimental treatments (Figure 4B and Supplementary Table S3). We analyzed immunoglobulins in plasma and cecal content as markers of the young rabbits' immune status. IgA relative concentration in cecal content was the highest at 18 days (*P* < 0.05), probably due to passive immunity (Figure 4C). Indeed, relative IgA concentration in cecal content dropped as milk ingestion decreased and reached the lowest level at day 38 (when rabbits eat only solid feed). Cecal IgA concentration then increased at day 57 (*P* < 0.05), probably in association with the development of the young rabbits' adaptive immune system. IgA levels were affected by the interaction age^*^experimental group (*P* = 0.009), with effects visible as of 29 days of age (Figure 4D). At 29 and 57 days, cecal IgA levels were significantly higher for rabbits from the FOS_GEL group compared to the AF_GEL group. Similarly, higher cecal IgA concentrations were found for the FOS_GEL group compared to the MOS_GEL group at 57 days. Plasma levels of IgG varied with age (*P* < 0.001) but were similar between groups (Figure 4E). Similarly to relative cecal IgA concentrations, IgG plasma levels were the highest at 18 days of age and reached the lowest level at days 29–38 before an increase at day 57. Overall, our results show that the stimulation of early-life solid feed ingestion by the supply of starter feed gels without an additive had no effect on the growth and health status of young rabbits. In contrast, supplementation of the starter feed gel with the FOS prebiotic increased IgA concentration in the cecum.

### Effects of Starter Feed Gels and Prebiotic Supplementation on the Microbiota of Young Rabbits

**Figure 5:**
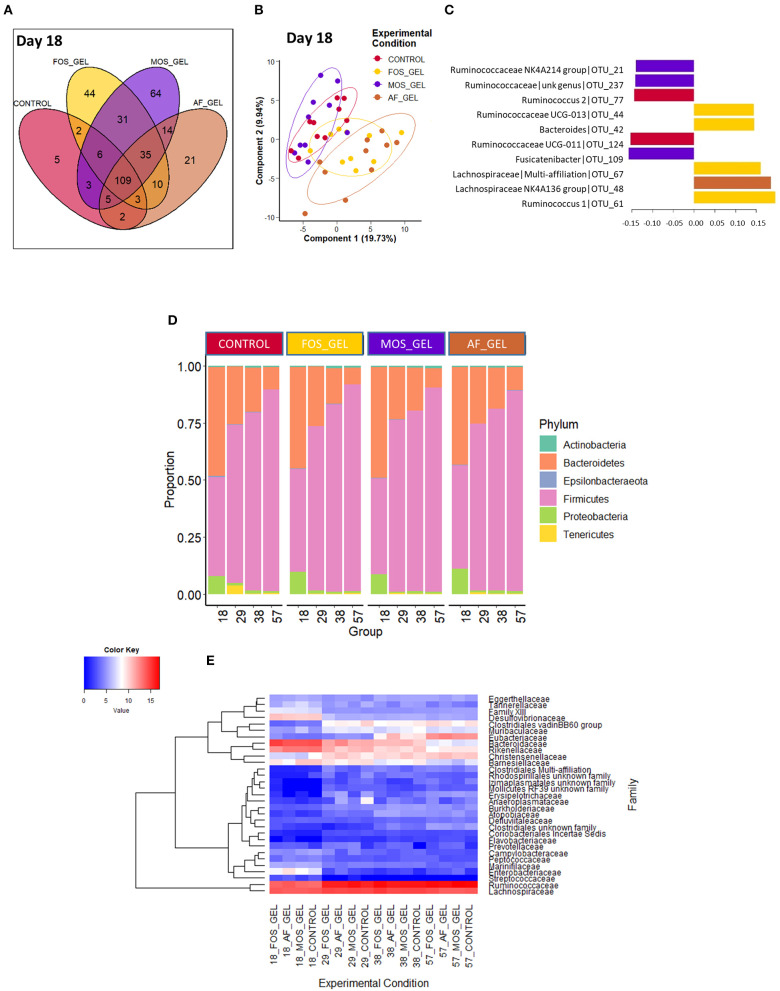
Venn diagram **(A)** occurrence of the OTUs across the cecal microbiota of 18-day-old rabbits according to early nutritional intervention in the form of a starter gel feed. The OTUs present in at least 75% of the samples within each group were kept to build the diagram. Partial least square discriminant analysis (PLS-DA, **B,C**) at the onset of early solid feed ingestion (d18) to classify samples according to the experimental group; 80% confidence ellipses are represented **(B)**. Loadings of the 10 most discriminant OTUs according to component 1 **(C)**. Phylum **(D)** and family **(E)** distributions according to early feed intake level. The heatmap **(E)** was built with rarefied count data after fourth root transformation. The additive composition of the gels varied according to treatments (FOS_GEL, fructo-oligosaccharides; MOS_GEL, a mixture of mannan-oligosaccharides and β-glucans; AF_GEL, no additive in the starter feed gel; CONTROL, no starter feed gel provided).

We evaluated the effects of starter feed gel intake and prebiotics on gut microbiota diversity and composition in young rabbits. In addition to the expected increase of observed OTUs and diversity indexes (Shannon and InvSimpson) with age, no significant difference could be seen regarding early nutritional intervention at the different time points of the analysis (Table 2). For analyses at the OTU level, we focused on the earliest time point (i.e., day 18) corresponding to the end of the gel supply. A Venn diagram was built to represent the shared OTUs in each experimental group of 18-day-old rabbits based on the OTUs present in at least 75% of individuals of each group, e.g., representative of the core bacterial community at this age (Figure 5A). Among the 354 OTUs studied, 31% were shared among all the rabbit cecal ecosystems, which accounted for 82% of total relative abundances. Only five OTUs were specific to the CONTROL group, while the other groups exhibited more specific taxa patterns. Animals from FOS_GEL and MOS_GEL groups had 12 and 18% of specific OTUs, respectively, but the latter only represented 0.4 and 0.9% of total relative abundances, respectively. The 35 OTUs that were shared between groups with early nutritional interventions (FOS_GEL, MOS_GEL, and AF_GEL) accounted for 6% of relative abundances. As for the OTUs not present in the CONTROL group (219 OTUs), they accounted for 12% of total relative abundances. A PLS-DA procedure was performed to determine if experimental groups had their own microbial composition signature based on OTUs with relative abundances >0.5% (Figure 5B). The PLS-DA individual plot at 18 days suggested two clusters: CONTROL/MOS_GEL and FOS_GEL/AF_GEL (Figure 5B). Among the discriminant OTUs, OTU_61, affiliated to the *Ruminococcus* genus, was significantly higher in the FOS_GEL group than in the MOS_GEL group (*P* < 0.05), while OTU_48, affiliated to the Lachnospiraceae NK4A136 group, had a higher level in the AF_GEL than in the CONTROL and MOS_GEL groups (Figure 5C and Supplementary Figure S1). As expected, OTU taxonomic assignation revealed that the shift in gut microbiota composition was coincident with aging and increased levels of solid feed in rabbit diets, with a progressive decrease in Bacteroidetes and Proteobacteria phyla (resp. from 46 and 9% at 18 days to 9 and 1% at 57 days) and a sharp increase in the Firmicutes phylum (from 44% at 18 days to 89% at 57 days) (Figure 5D). The Tenericutes phylum tended to be affected by the experimental group (*P* = 0.08). A total of 99.5 and 68.1% of the sequences could be assigned at the family and genus levels, respectively. The proportions of families with relative abundances >0.5% were similar between the four experimental groups (Figure 5E). Overall, our results show that the stimulation of early life solid feed ingestion by starter feed gels had no effect on alpha diversity but seemed to allow the implantation of specific OTUs, some of them related to the prebiotic supplementation. However, their abundances in the ecosystems remained moderate.

**Table 2:** Effects of early nutritional interventions on the alpha-diversity metrics of rabbit microbial cecum ecosystems (mean ± standard deviation).

**Age (days)**	**Experimental group**	**Number of observed OTUs**	**Shannon index**	**InvSimpson index**
18	CONTROL	259 ± 52	3.47 ± 0.35	16.1 ± 6.0
	FOS_GEL	272 ± 64	3.61 ± 0.28	17.2 ± 4.6
	MOS_GEL	248 ± 53	3.44 ± 0.31	14.5 ± 4.3
	AF_GEL	259 ± 67	3.42 ± 0.42	14.1 ± 6.2
29	CONTROL	411 ± 110	4.21 ± 0.59	30.2 ± 11.6
	FOS_GEL	418 ± 71	4.47 ± 0.21	38.8 ± 9.3
	MOS_GEL	444 ± 64	4.57 ± 0.26	44.6 ± 10.9
	AF_GEL	425 ± 83	4.39 ± 0.43	36.5 ± 13.0
38	CONTROL	489 ± 89	4.68 ± 0.27	48.0 ± 13.9
	FOS_GEL	468 ± 105	4.58 ± 0.47	47.8 ± 28.9
	MOS_GEL	530 ± 95	4.77 ± 0.33	52.6 ± 16.8
	AF_GEL	506 ± 65	4.80 ± 0.21	55.6 ± 17.8
57	CONTROL	593 ± 46	5.04 ± 0.19	66.4 ± 16.3
	FOS_GEL	579 ± 59	5.11 ± 0.14	73.4 ± 14.8
	MOS_GEL	596 ± 55	5.07 ± 0.20	64.8 ± 17.4
	AF_GEL	581 ± 52	5.12 ± 0.14	75.0 ± 12.7
***P-*****value**				
Age		<0.001	<0.001	<0.001
Group		0.926	0.759	0.691
Age * Group		0.616	0.326	0.125

*The additive composition of the starter feed gels varied according to treatments (CONTROL, no starter feed gel provided; FOS, fructo-oligosaccharides; MOS, a mixture of mannan-oligosaccharides and β-glucans; AF, no additive in the starter feed gel)*.

### Effects of the Level of Early-Life Solid Feed Ingestion on the Microbiota of Young Rabbits

Based on our observation that the MOS_GEL group had the lowest solid feed intake compared to the FOS_GEL and AF_GEL groups and that its microbiota composition clustered with that of the CONTROL group with no gel supplementation, we hypothesized that ingestion levels in the nest were a structural factor of bacterial community implantation rather than our prebiotic supplementation. Consequently, we added a new categorial variable in our analysis based on the total starter feed intake in the nest of the litters sampled: it was either null (Null group, *n* = 10 litters), below the median (<3.8 g of fresh gel consumed/rabbit; *n* = 15), or above the median (>3.8 g; *n* = 15). One litter from the MOS_GEL group, five from the AF_GEL group and nine from the FOS_GEL group were classified with high early feed intake (“above the median”) (Supplementary Figure S2). Pellet intake in feeders was also the highest in rabbits from the “above the median” group at 18 and 21 days (+15 g of dry matter from 15 to 21 days) (Supplementary Figure S3).

**Figure 6:**
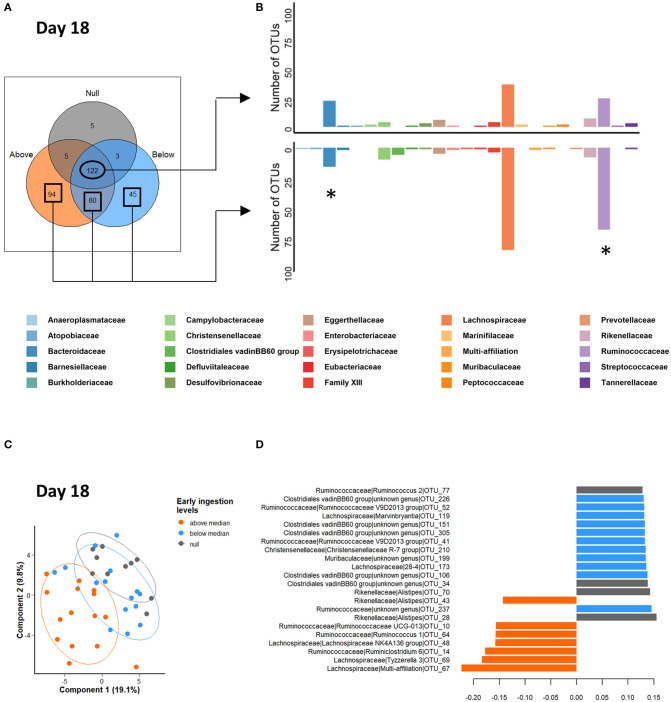
Venn diagram **(A)** occurrence of the OTUs across the cecal microbiota of 18-day-old rabbits according to starter feed gel ingestion level. The OTUs present in at least 75% of the samples within each group were kept to build the diagram. **(B)** Distribution among bacterial family affiliations of the OTUs shared by all the rabbits (upper barplot) and by the rabbits with a starter gel feed ingestion in the nest (lower barplot). The stars represent significant differences of Bacteroidaceae and Ruminococcaceae distributions. **(C,D)** Partial least square discriminant analysis at the onset of early solid feed ingestion (d18) to classify samples according to starter gel feed ingestion level; 80% confidence ellipses are represented **(C)**. Loadings of the 22 discriminant OTUs according to component 1 **(D)**. Null group: no feed intake corresponding to the 10 CONTROL group litters; “Below median group”: with an intake under 3.8 g of fresh gel consumed/rabbit (*n* = 15 litters); and “Above median” group: an intake over 3.8 g of fresh gel consumed/rabbit (*n* = 15 litters).

A new Venn diagram was built based on the OTUs present in at least 75% of individuals in the three intake level groups (Figure 6A). This diagram clearly emphasized the presence of specific OTUs in relation to early solid feed ingestion. They represented 219 OTUs out of 354, with a higher number of OTUs belonging to the Ruminococcaceae family and a lower number belonging to the Bacteroidaceae family compared to the shared OTUs (χ^2^ = 4.95, *df* = 1, *P* = 0.026, and χ^2^ = 7.26, *df* = 1, *P* = 0.007, respectively; Figure 6B). Cumulated specific OTUs of the “above median” and “below median” groups represented 4.2 and 0.5%, respectively, of total relative abundances (Supplementary Figure S4). Subsequent PLS-DA two-component projections at 18 days (Figure 6C) suggested that individuals that consumed the highest quantities of gel in the nest are clustered in a separate group. Among the 22 discriminant OTUs (Figure 6D), OTU_67 and OTU_14, affiliated to the Lachnospiraceae family and the *Ruminoclostridium 6* genus, respectively, exhibited the highest relative abundances in rabbits with the highest starter feed gel ingestion level in the nest (“above median” group) (Supplementary Figure S5). This clustering pattern was also observable at 29 days of age (Supplemental Figure S6). Interestingly, several days after weaning, the levels of early feed ingestion still affected microbiota composition. Indeed, supervised multivariate analysis at 38 and 57 days (Supplementary Figure S6) revealed that the three intake level groups still exhibited differences in their microbial communities. However, as of 29 days of age, PLS-DA total explained variance was low (≃10% on the two first components).

**Figure 7:**
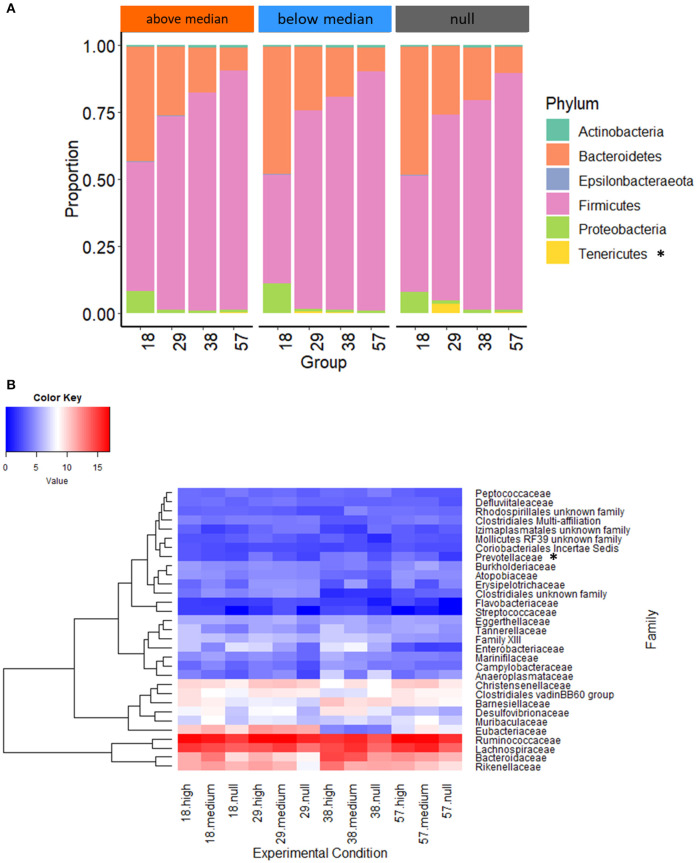
Phylum **(A)** and family **(B)** distributions according to early feed intake level. The heatmap **(B)** was built with rarefied count data after fourth root transformation. Null group: no feed intake corresponding to the 10 CONTROL group litters: “Below median” group: with an intake under 3.8 g of fresh gel consumed/rabbit (*n* = 15 litters); and “Above median” group: an intake over 3.8 g of fresh gel consumed/rabbit (*n* = 15 litters). Phyla or family with significant differences of distribution between experimental groups at one sampling date are followed by an asterisk.

Analysis of the taxonomic assignation of OTUs revealed that the abundances of the Tenericutes phylum in the cecum of 29-day-old rabbits with no previous access to starter feed gels was 36 and 6 times greater than for rabbits with high (*P* < 0.001) and intermediate early feed intake (*P* = 0.02), respectively (Figure 7A). The distribution of the 32 taxonomic families in the rabbit cecum was similar regardless of early feed ingestion (Figure 7B) except for Prevotellaceae, which was more abundant at 29 days (*P* = 0.009) when suckling rabbits consumed large quantities of gels. At the genus level, only the *Paraprevotella* genus was affected by early feed intake levels, with a greater abundance in rabbits from the “above median” intake group (*P* = 0.02). Altogether, our results show that starter gel feed intake level was a better explanatory factor of differential microbial compositions up to 57 days than the original experimental groups. When rabbits consumed more starter feed, the highest proportions of bacteria with plant-degrading abilities, such as species from the Lachnospiraceae and Ruminococcaceae families, were observed.

### Cecal Activity and Gut Morphology

No differences were observed between prebiotic supplementation groups or early feed intake level groups on cecal fermentative activity at 29, 38, and 57 days (Table 3). Gut characteristics, determined with cecum, cecal digesta, stomach, and intestine weights, did not vary according to prebiotic supplementation groups or early feed intake level groups (Supplementary Table S4).

**Table 3:** Influence of early dietary intervention and age on cecal fermentation characteristics.

**Age** **(days)**	**Experimental group**	**pH**	**NH3** **mM**	**Total VFA** **mM**	**Acetate** **%**	**Propionate** **%**	**Butyrate** **%**	**Ratio C3/C4^**a**^**
29	CONTROL	5.9	20.0	104	85.9	5.6	8.8	1.10
	FOS_GEL	5.9	17.1	107	86.0	4.2	9.8	0.51
	MOS_GEL	5.8	15.4	118	86.0	3.9	10.2	0.41
	AF_GEL	5.9	20.1	104	87.3	4.3	8.4	0.58
38	CONTROL	5.8	4.3	126	87.9	3.7	8.5	0.47
	FOS_GEL	5.7	3.7	134	88.2	3.7	8.2	0.48
	MOS_GEL	5.8	5.0	124	88.3	4.1	7.7	0.75
	AF_GEL	5.7	3.4	128	88.1	3.4	8.5	0.42
57	CONTROL	5.6	3.4	146	83.6	3.2	13.2	0.25
	FOS_GEL	5.5	2.8	152	85.9	3.0	11.1	0.28
	MOS_GEL	5.7	3.4	141	85.3	3.3	11.4	0.30
	AF_GEL	5.4	4.1	156	84.2	3.2	12.7	0.26
***P-*****value**								
SEM^b^		0.5	2.9	43	1.0	0.6	1.0	0.3
Age		<0.001	<0.001	<0.001	<0.001	<0.001	<0.001	<0.001
Group		0.139	0.304	0.327	0.517	0.790	0.556	0.972
Age * Group		0.221	0.502	0.244	0.191	0.791	0.108	0.248

*The additive composition of the starter feed gels varied according to treatments (CONTROL, no starter feed gel provided; FOS, fructo-oligosaccharides; MOS, a mixture of mannan-oligosaccharides and β-glucans; AF, no additive in the starter feed gel)*.

a *Ratio (C3/C4): propionate/butyrate ratio*.

b *SEM, Standard Error of the Mean*.

## Discussion

In young mammals, progressive implantation of the digestive microbiota initiates the microbiota-host dialogue that conditions the development and stimulation of the immune system (8, 47). This process is largely impacted by the substrate that arrives in the gut. The weaning transition, i.e., the dietary transition from a milk-based diet to a solid diet, shifts the microbial balance and contributes to digestive maturation (15, 16, 36). Managing this feed transition is therefore crucial for the construction of the animal's health and its future preservation. The purpose of our study was, on the one hand, to assess the effects of stimulation of early solid feed intake on rabbit microbiota establishment and, on the other, to determine if two selected prebiotics can be relevant tools to manipulate the developing microbiota. We demonstrated that the quantity of feed consumed in early life was a stronger driver of microbiota implantation than the nature of the prebiotic supplementation.

### Stimulation of Early Feed Intake: Consequences on Rabbit Growth and Health

In most mammal organisms, the onset of a solid diet intake depends on their ability to exhibit feed prehension motor, mastication, and swallowing patterns (48). The beginning of solid feed intake is variable between species: for example, guinea pigs are able to process hard feed as of 1 day of age, whereas feeding activities begin at 18 days in rat pups (49). In commercial rabbit farms, rabbits start to consume feed at around 17–20 days, once they are mobile enough to leave the nest (50). However, rabbits are able to ingest solid substrates at an earlier age: wild rabbits are known to consume nest material 8 days after birth (17), and in a situation of choice between pellets differing in size or hardness, pups consumed them as of 8 days of age and expressed dietary preferences (21). Considering previous results on young rabbit feeding behavior (29), we designed a starter feed in a gel form that was rich in moisture and adapted to the suckling rabbit's physiological and physical constraints. It was supplemented with attractive vanilla flavor to take advantage of the fully-developed olfactory system of rabbit neonates (51, 52). This starter feed gel was provided in the nest with easy access in order to mimic the feed nibbling behavior observed in wild conditions. We opted to include prebiotics in the experimental starter feed based on their relevance to modify rabbit microbiota (23, 53) and phenotypic traits (54, 55), and based on the presence of oligosaccharides in milk as an essential driver of gastrointestinal microbiota development (56). Consequently, in one of the experimental groups, we provided FOS, which are known to stimulate bifidobacteria and lactobacilli growth but can also target other species from the Actinobacteria phylum or *Olsenella* genus, for example (57). In another group, we distributed MOS, which were found to block enteric pathogen colonization in numerous studies (58), in addition to the immunomodulating β-glucans (59).

In good agreement with the early feed intake kinetics previously described (21), feed intake started as early as 7 days of age and exponentially increased over time. This study confirmed that birth weight was a determining factor of early solid feed intake (29). Interestingly, the nature of the gels provided affected their intake in the nest, with higher ingestion of gels containing FOS compared to MOS. Prebiotics are known to modify sensory feed characteristics, and FOS additives contribute to a sweet taste similar to that of sucrose (60). Knowing that rabbits are attracted by bitter and sweet aromas (61), this taste difference probably explains the greater attractiveness of FOS gels. The taste of MOS highly depends on the type of product (distillery, brewery by-products or primary grown yeast). The MOS product used in this study was a yeast cell wall with a low protein content processed from primary fermentation, and with no specific flavor-contributing properties. Another hypothesis to explain ingestion level differences due to prebiotic additives may be linked to their effects on hormone production (62). These authors demonstrated that prebiotic supplementation in healthy humans was associated with an increase in plasmatic gut peptide concentrations that reduced appetite sensations.

To our knowledge, this study is the first one to highlight variations in cecal IgA content as a function of prebiotic supplementation in rabbits, although it has been well-described in infants who were fed formula containing prebiotics (63). IgA are essential for gut homeostasis since they represent first-line mucosal defenses by providing non-inflammatory immune protection (64). Microbiota modulation induced by starter feed intake could explain the effects on IgA content observed after weaning since some bacteria can induce the development of isolated lymphoid follicles (65). Further experiments are now necessary to determine if the higher levels of IgA after early FOS intake are due to the prebiotics themselves or the higher level of solids ingested in this group.

### Attempts to Modulate Cecal Microbiota With Early Nutritional Interventions

When we tried to discriminate rabbit microbiota composition according to the prebiotic supplementation before weaning, we obtained unsuccessful partitioning. Nevertheless, when the analysis was performed by considering the variations in starter feed intake between litters rather than the nature of the prebiotics provided, better patterns were evidenced. Limited effects of the prebiotics used can be explained by the specificity of the suckling rabbit microbiota. Indeed, in the first 2 weeks of life, bacterial density and diversity in the cecum are low compared to older rabbits (10). Lactobacilli, a potential target of FOS, are rare inhabitants of the digestive tract in rabbits and, even if their growth is stimulated, they poorly adhere to rabbit intestinal epithelial cells (18, 66). Moreover, we did not infect the animals with enteric pathogens during this study whose sanitary conditions were good, which could explain why MOS effects on the adhesion of enteropathogenic bacteria were not evidenced. Thus, the young rabbit gut may not be a relevant bacterial reservoir for the prebiotics used, which need to target specific indigenous microorganisms to be effective. Moreover, it should be pointed out that rabbit diets are naturally rich in fibrous ingredients, some of them having significant amounts of oligosaccharides (67), which could have hidden effects of prebiotic treatments.

Early-life solid feed consumption was associated with 219 specific OTUs at 18 days of age, which mainly belonged to the Lachnospiraceae and Ruminococcaceae families. Members of those families are efficient fermenters of complex plant materials such as cellulose and have been associated with gut health maintenance (68). The role of some Lachnospiraceae strains in butyrate production was demonstrated *in vivo*, a function that can have subsequent protective health effects, notably through colonic Treg induction (69). We observed that high proportions of the *Paraprevotella* genus were also associated with high consumption of starter feeds. One of the two species described from this genus can break down the xylans in plant cell walls to produce succinate and acetate (70). Taken together, those findings may indicate that the small amounts of feed ingested at the onset of a solid diet are sufficient to prepare the weaning transition by selecting bacteria able to ferment plant-based diets with end products such as butyrate, a well-known promoter of the epithelial barrier (71).

It was observed that an early nutritional intervention had long-term consequences with differential microbiota structures up to 39 days afterwards. We can hypothesize that those subsequent modifications are related to the increase of pellet intake in the feeders during the middle nursing period, induced by greater starter feed consumption. This phenomenon has been well-described in pig farming systems (72) where suckling pigs offered starter feed in a liquid or gruel form in addition to milk increased pellet intake later during the nursing period. Since a similar phenomenon was observed in suckling rabbits (29) and given the impact of solid feed on bacterial community structures (15, 73), this posterior effect of early feed intake stimulation on feeding behavior appears interesting and should be further explored.

Despite some changes in cecal ecosystems induced by the amount of starter feed that reached the gut, it is worthwhile noting that no changes in cecal fermentation activity were observed. The reason for this lack of functional modifications could be the functional redundancy within the microbiome since different bacterial populations within a community can perform the same functions (74). The fact that alpha and beta diversity were not modified by early feed intake stimulation and that limited effects on taxonomic profiles were revealed could also be possible explanations for the lack of functional changes in the cecum. It is also likely that few effects were observed due to predominant milk intake in the young rabbit's diet up to 25–30 days (50). In a previous experiment, we estimated that a total pellet intake of 1.8 g of fresh matter in the nest from 8 to 17 days only amounted to 1.3% of the total milk intake over this period (21). Thus, large amounts of milk may have “diluted” the effects of solid supplementation since rabbit milk constrains bacterial community structures (36). Indeed, mammals' milk contains its own microbiota, prebiotics (oligosaccharides), immunoglobulins, and other microbiota-shaping compounds such as antimicrobial casein-derived peptides or lipids (75, 76) that probably induce a selective pressure. This dominant impact of milk consumption was shown in human cohort studies where the duration of exclusive breastfeeding was a stronger driver for microbial diversity at 9 months of age than the time when solid food was introduced into the infant's diet (77). In accordance, previous attempts to modulate rabbit microbiota before weaning with diet change (36, 78); had moderate effects, possibly due to milk constraint. Another possible explanation for early dietary intervention limitations is the heterogeneity of the solid intake in the nest within litters. Competition for feed might have occurred between rabbits, as observed during nursing (79), and similarly with pigs whose variability in individual starter feed intake was demonstrated with a stool marker (80). In our trial, rabbits were randomly sampled due to the absence of a methodology to determine “good eater” rabbit up until now.

## Conclusion

Starter feed provided in a gel form was accepted by suckling rabbit pups with intake differences depending on the type of gels offered. Gels preferably consumed in the nest were associated with increased solid feed intake later in life. Supplementation with prebiotics in early life did not have a notable effect on the gut microbiota of suckling rabbits before and after weaning. When considering the amounts of starter feed ingested instead of the nature of the prebiotics consumed, more pronounced effects on bacterial composition could be observed. Increased quantities of feed consumed at an early age seemed to promote the development of microorganisms adapted to plant degradation, which could efficiently prepare the rabbits for weaning transition. Further studies are now necessary to identify optimal gut-shaping starter feeds and to confirm subsequent effects on ecosystem functionalities and gut health.

## Data Availability Statement

All raw sequences were deposited in the NCBI Sequence Read Archive (SRA) under accession number PRJNA589727.

## Ethics Statement

This animal study was reviewed and approved by Science et Sante Animales No. 115, Ecole Nationale Vétérinaire, France.

## Author Contributions

CP, TG, KB, JD, CG, EG-G, GR, and SC conceived and designed the experiments. CP, OB, CB, and PA carried out and planned the experiments. CP and SC analyzed the data and wrote the manuscript.

## Conflict of Interest

CP was employed by the company CCPA for the Groupe d'Experimentation Cunicole network (French network of companies that includes CCPA, EVIALIS, MiXscience, TECHNA, and Wisium). KB was employed by the company CCPA. JD was employed by the company EVIALIS. CG was employed by the company MiXscience. GR was employed by the company TECHNA. EG-G was employed by the company Wisium. The remaining authors declare that the research was conducted in the absence of any commercial or financial relationships that could be construed as a potential conflict of interest.

## Abbreviations

AF: additive-free
DM: dry matter
FOS: fructo-oligosaccharides
MOS: mannan-oligosaccharides
OUT: operational taxonomic units
PLS-DA: partial least squares discriminant analysis
VFA: volatile fatty acids.
