# Supplementary material for: Early Introduction of Solid Feeds: Ingestion Level Matters More Than Prebiotic Supplementation for Shaping Gut Microbiota
Source: Front Vet Sci. 2020 May 15;7:261. doi: 10.3389/fvets.2020.00261 (PMC7242618; doi:10.3389/fvets.2020.00261)
Supplement: Supplementary file 1 [file Data_Sheet_1.docx]

Supplementary Material

Early introduction of solid foods: ingestion level matters more than prebiotic supplementation for shaping gut microbiota

# Supplementary Figures and Tables

## Supplementary Figures

**Supplementary Figure S1:** Relative abundance of the top ten discriminant OTUs at 18 days of age (based on PLS-DA analysis) according to early nutritional intervention. The additive composition of the gels varied according to treatments (CONTROL: no starter food gel provided; FOS: fructo-oligosaccharides; MOS: a mixture of mannan-oligosaccharides and β-glucanes; AF: no additive in the starter food gel). Mean ± standard deviation.


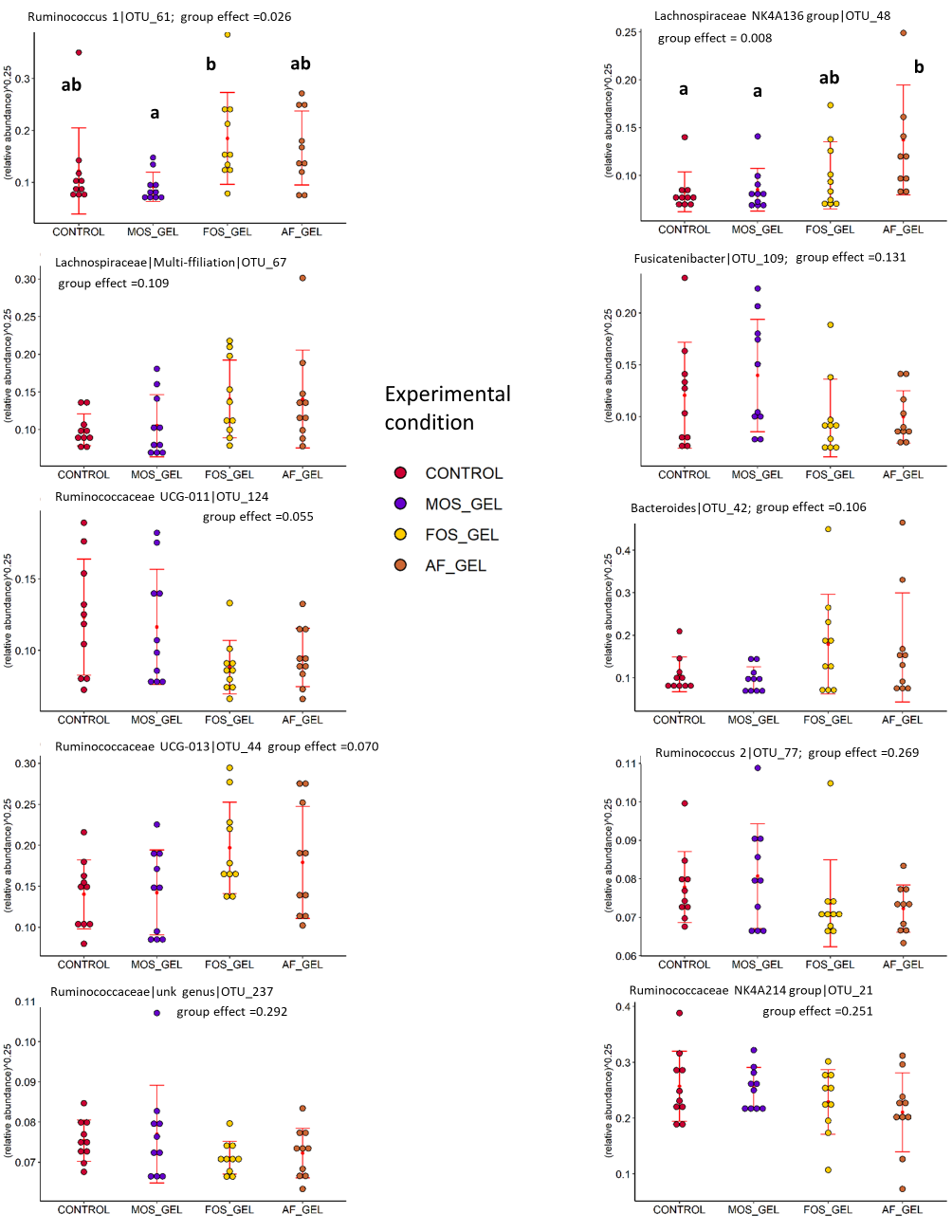


**Supplementary Figure S2**: Distribution of the litters according to their total starter food gel intake and their early nutritional intervention **A.** Total intake in the nest of starter food gel (FOS_GEL: gel with fructo-oligosaccharides additive; MOS_GEL gel with a mixture of mannan-oligosaccharides and β-glucanes; AF_GEL: no additive in the starter food gel). **B.** Origin of the litters according to early nutritional intervention (row) and their distribution according to the new categorial variable based on the total starter food intake in the nest (Null group: no food intake corresponding to the ten CONTROL group litters; “Below median” group: with an intake under 3.8 g of fresh gel consumed/rabbit (n=15 litters); and “Above median” group: an intake over 3.8 g of fresh gel consumed/rabbit (n=15 litters)).

**A.** **B.**

| 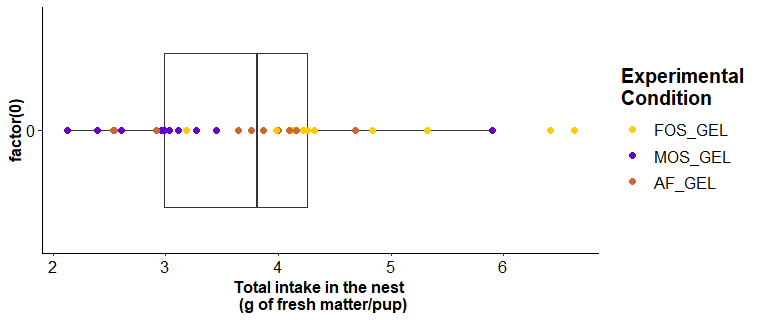 |  | **Above median** | **Below median** | **Null** |
| --- | --- | --- | --- | --- |
|  | **CONTROL** | 0/15 | 0/15 | 10/10 |
|  | **FOS_GEL** | 9/15 | 1/15 | 0/10 |
|  | **MOS_GEL** | 1/15 | 9/15 | 0/10 |
|  | **AF_GEL** | 5/15 | 5/15 | 0/10 |

**Supplementary Figure S3:** Long-term effects of early food intake levels on subsequent pellet intake before weaning.

Null group: no food intake corresponding to the ten CONTROL group litters; “Below median” group: with an intake under 3.8 g of fresh gel consumed/rabbit (n=15 litters); and “Above median” group: an intake over 3.8 g of fresh gel consumed/rabbit (n=15 litters). Significant differences between experimental groups found within ages are represented with different letters.


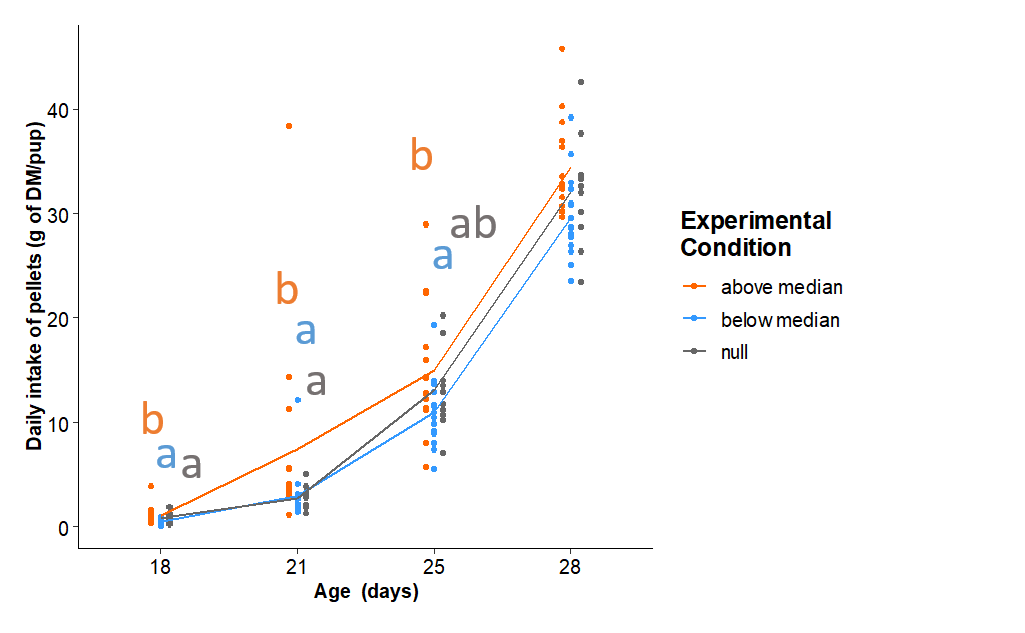


**Supplementary Figure S4:** Relative abundance of the 219 OTUs shared by at least 75% of the rabbits provided with early food gels in the nest.

**
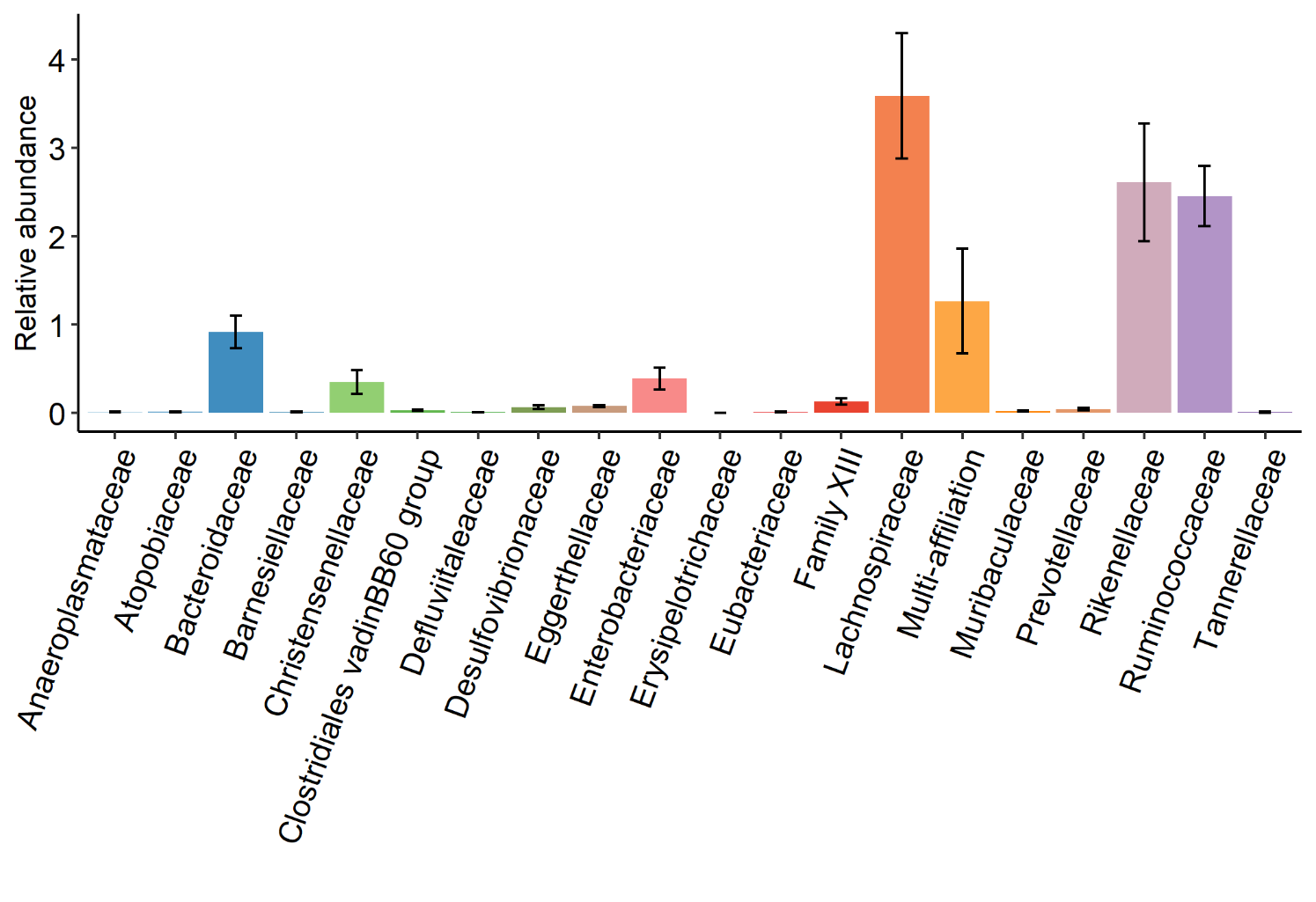
**

**Supplementary Figure S5:** Relative abundance of the top ten discriminant OTUs at 18 days of age according to starter food gel ingestion level.

Null group: no food intake corresponding to the ten CONTROL group litters; “Below median” group: with an intake under 3.8 g of fresh gel consumed/rabbit (n=15 litters); and “Above median” group: an intake over 3.8 g of fresh gel consumed/rabbit (n=15 litters).


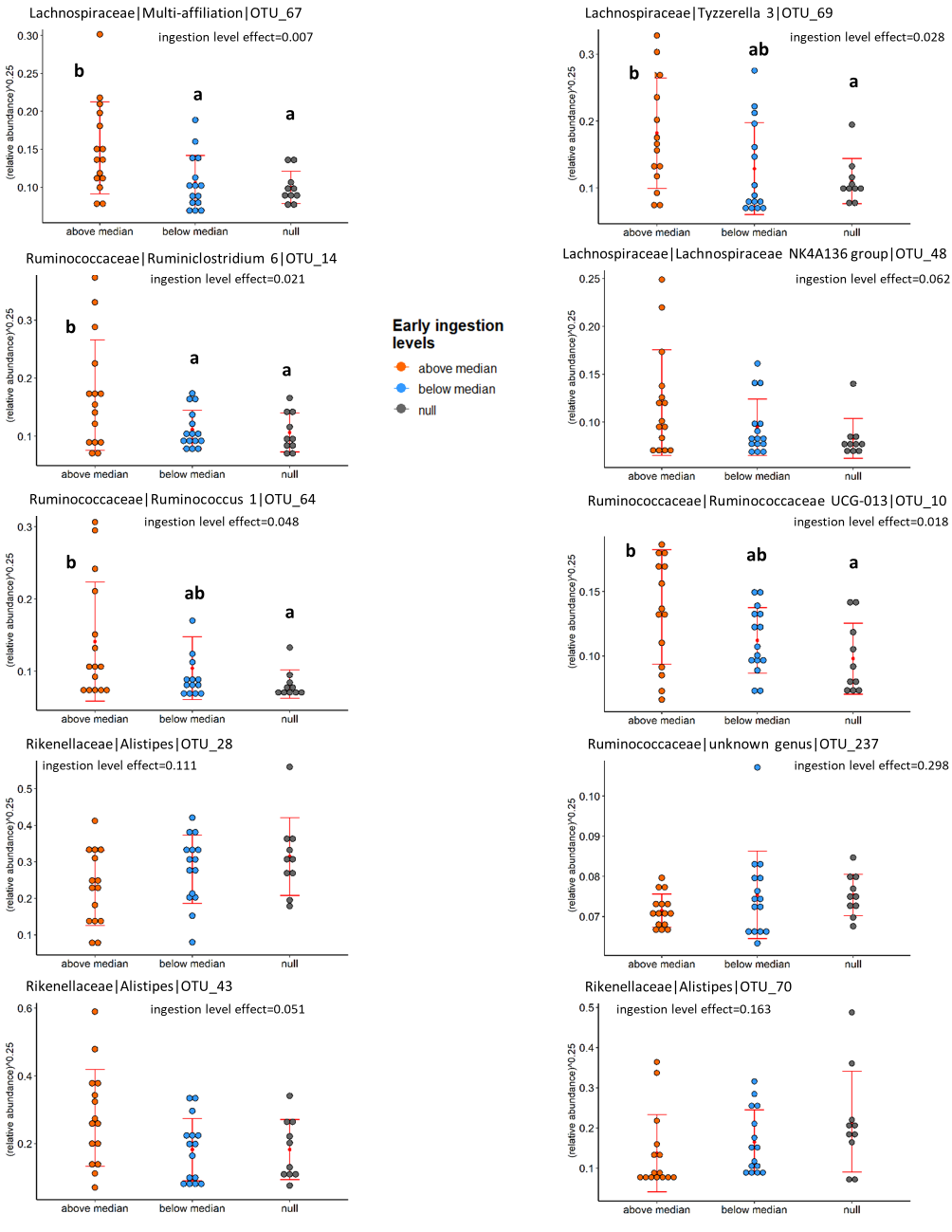


**Supplementary Figure S6:** Partial least square discriminant analysis at d29, d38 and d57 according to experimental groups **(A)** or based on early food intake levels **(B)**.

CONTROL: no starter food gel provided; FOS_GEL: gel with fructo-oligosaccharides additive; MOS_GEL gel with a mixture of mannan-oligosaccharides and β-glucanes; AF_GEL: no additive in the starter food gel.

Null group: no food intake corresponding to the ten CONTROL group litters; “Below median” group: with an intake under 3.8 g of fresh gel consumed/rabbit (n=15 litters); and “Above median” group: an intake over 3.8 g of fresh gel consumed/rabbit (n=15 litters).

| **A.**  **B.** | **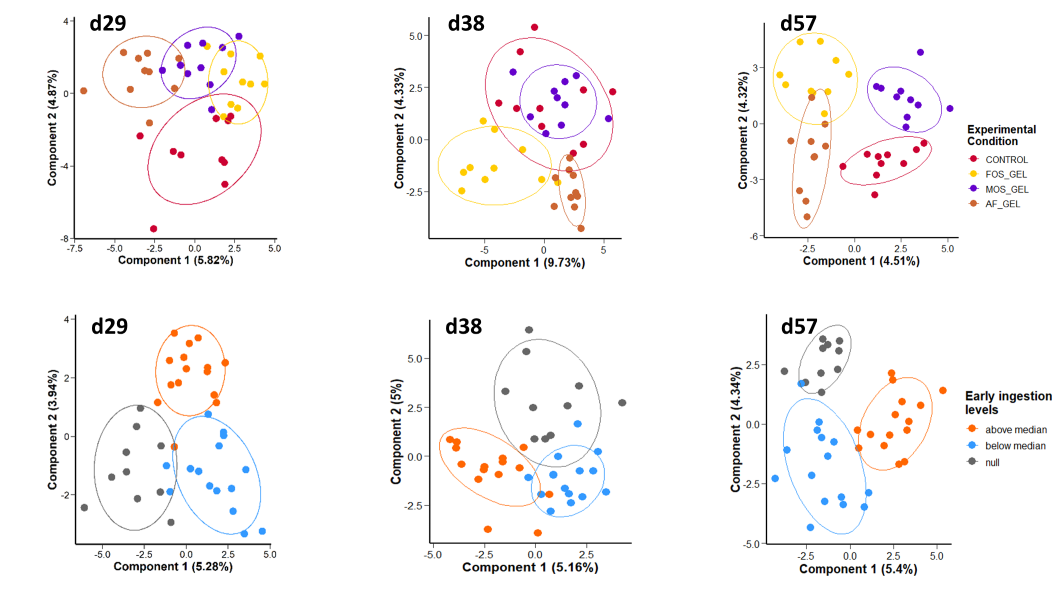** |
| --- | --- |

## Supplementary Tables

**Supplementary Table S1**| Formulas to describe the statistical models used on R.

| **Partial Least Squares Discriminant Analysis** | > Package mixOmics (version 6.6.0)  *plsda(OTU, Group, ncomp=3, logratio=CLR)*  *OTU: OTUs counts after Total Sum Scaling normalization*  *Group: experimental groups or early feed intake level* |
| --- | --- |
| **Linear mixed models with correction for age heteroscedasticity** | *>* Package nlme (version 3.1)  *lme(Response variable1) ~ ((Day * Group, random=~1\|Litter), weights=varIdent(form=~1\|Day))*  *Response variable1: daily feed intake, growth data, cecal and blood parameters*  *Group: experimental groups or early feed intake level* |
| **Linear mixed models** | *>* Package nlme (version 3.1)  *lme(Response variable2) ~ Day * Group, random=~1\|Litter*  *Response variable2: taxonomic relative abundances after square root transformation, diversity indexes*  *Group: experimental groups or early feed intake level* |
| **Analysis of covariance** | > Package car (version 3.0)  *lm(Total gel consumption) ~ Group + Nest score + Litter weight at equalization*  *Total gel consumption ~ N(0, σ²)*  *Group: experimental groups or early feed intake level*  *Litter weight at equalization: quantitative covariate* |
| **Adjusted *chi-squared*** | > Package aod (version 1.3)  *donner(cbind(deaths, N-deaths) ~ Group)*  *Deaths: number of dead pups for each litter*  *N: number of pups at the beginning of the experiment for each litter*  *Group: experimental groups or early feed intake level* |

**Supplementary Table S2**| Effects of early nutritional interventions on subsequent intake of pre-weaning pellets (mean ± standard deviation). Different letters stand for significant differences revealed after multiple paired comparisons with Bonferroni corrections. CONTROL: no starter food gel provided; FOS: fructo-oligosaccharides; MOS: a mixture of mannan-oligosaccharides and β-glucanes; AF: no additive in the starter food gel.

|  | **CONTROL** | **FOS_GEL** | **MOS_GEL** | **AF_GEL** |
| --- | --- | --- | --- | --- |
| **Food intake (g/pup/day)** | | | | |
| 15-18 days | 0.8 ± 0.5 ^ab^ | 1.2 ± 1.0 ^b^ | 0.6 ± 0.4 ^a^ | 0.6 ± 0.3 ^a^ |
| 18-21 days | 2.6 ± 1.2 ^a^ | 8.4 ± 11.1 ^b^ | 4.3 ± 4.1 ^a^ | 2.9 ± 1.1 ^a^ |
| 21-25 days | 12.8 ± 3.8 | 16.7 ± 6.3 | 10.9 ± 4.8 | 12.2 ± 2.2 |
| 25-28 days | 31.8 ± 5.3 | 34.7 ± 5.4 | 30.4 ± 5.1 | 31.9 ± 4.0 |
| 28-32 days | 46.0 ± 7.4 | 44.9 ± 4.7 | 43.2 ± 5.5 | 42.3 ± 8.5 |
| 32-35 days | 51.5 ± 15.6 | 57.0 ± 17.1 | 55.4 ± 16.5 | 5.7 ± 15.8 |
| *Age effect: P<0.001* | |  |  |  |
| *Group effect: P=0.059* | |  |  |  |
| *Age * Group effect: P=0.014* | |  |  |  |

**Supplementary Table S3**| Early nutritional interventions did not affect growth performances before and after weaning. Values are mean ± standard deviation.

|  | **CONTROL** | **FOS_GEL** | **MOS_GEL** | **AF_GEL** |
| --- | --- | --- | --- | --- |
| **Weight before weaning (g/rabbit)** | | | | |
| 3 days | 98 ± 4 | 101 ± 8 | 95 ± 16 | 99 ± 10 |
| 14 days | 293 ± 19 | 291 ± 32 | 303 ± 20 | 296 ± 21 |
| 21 days | 411 ± 32 | 403 ± 60 | 437 ± 32 | 413 ± 46 |
| 28 days | 698 ± 38 | 697 ± 65 | 707 ± 33 | 704 ± 44 |
| 35 days | 1009 ± 104 | 993 ± 129 | 1028 ± 111 | 1027 ± 110 |
| *Age effect: P<0.001* | |  |  |  |
| *Group effect: P=0.735* | |  |  |  |
| *Age * Group effect: P=0.423* | |  |  |  |
| **Weight after weaning (g/rabbit)** | | | | |
| 50 days | 1665 ± 150 | 1666 ± 208 | 1656 ± 215 | 1709 ± 111 |
| 64 days | 2233 ± 173 | 2236 ± 290 | 2245 ± 273 | 2279 ± 131 |
| *Age effect: P<0.001* | |  |  |  |
| *Group effect: P=0.666* | |  |  |  |
| *Sex effect: P=0.627* | |  |  |  |
| *Age * Group effect: P=0.020* | | | | |
| **Average daily weight gain before weaning (g/day)** | | | | |
| 3 – 21 days | 17.4 ± 1.7 | 16.8 ± 3.3 | 19.0 ± 1.7 | 17.5 ± 2.5 |
| *Group effect: P=0.174* | |  |  |  |
| 21 – 35 days | 42.9 ± 3.0 | 42.8 ± 3.5 | 42.3 ± 3.5 | 43.7 ± 3.1 |
| *Group effect: P=0.790* | |  |  |  |
| **Average daily weight gain after weaning (g/day)** | | | | |
| 35 – 50 days | 44.0 ± 6.9 | 44.6 ± 8.0 | 41.5 ± 11.4 | 45.1 ± 5.0 |
| *Group effect: P=0.248* | |  |  |  |
| *Sex effect: P=0.999* | |  |  |  |
| 35 – 64 days | 43.6 ± 6.1 | 42.9 ± 7.5 | 44.1 ± 7.5 | 43.1 ± 5.4 |
| *Group effect: P=0.143* | |  |  |  |
| *Sex effect: P=0.808* | |  |  |  |

**Supplementary Table S4**| Early nutritional interventions did not modify the development of the gastrointestinal tract (mean ± standard deviation).

|  | **CONTROL** | **FOS_GEL** | **MOS_GEL** | **AF_GEL** |
| --- | --- | --- | --- | --- |
| **Cecum weight (tissue + digesta, in grams)** | | | | |
| 18 days | 4.8 ± 1.2 | 4.8 ± 1.6 | 5.4 ± 1.2 | 5.7 ± 1.1 |
| 29 days | 39.4 ± 9.4 | 35.2 ± 6.0 | 38.3 ± 7.6 | 37.8 ± 10.5 |
| 38 days | 82.0 ± 5.3 | 84.5 ± 11.0 | 83.0 ± 15.3 | 80.4 ± 6.9 |
| 57 days | 169.2 ± 24.1 | 156.8 ± 18.2 | 155.6 ± 26.4 | 174.4 ± 17.8 |
| *Age effect: P<0.001* | | | | |
| *Group effect: P=0.889* | | | | |
| *Age * Group effect: P=0.517* | | | | |
| **Cecum empty weight (g)** | | | | |
| 18 days | 3.2 ± 0.4 | 3.0 ± 0.5 | 3.2 ± 0.4 | 3.2 ± 0.3 |
| 29 days | 4.8 ± 1.4 | 4.3 ± 1.2 | 4.7 ± 1.2 | 4.7 ± 1.2 |
| 38 days | 18.4 ± 1.8 | 19.5 ± 1.7 | 18.8 ± 2.2 | 19.1 ± 2.6 |
| 57 days | 31.5 ± 3.7 | 30.1 ± 3.0 | 31.2 ± 4.5 | 30.7 ± 2.3 |
| *Age effect: P<0.001* | |  |  |  |
| *Group effect: P=0.859* | |  |  |  |
| *Age * Group effect: P=0.486* | |  |  |  |
| **Ratio cecum weight / body weight (%)** | | | | |
| 18 days | 1.3 ± 0.3 | 1.3 ± 0.3 | 1.3 ± 0.3 | 1.5 ± 0.3 |
| 29 days | 5.1 ± 1.3 | 4.4 ± 0.6 | 4.8 ± 0.8 | 4.9 ± 1.4 |
| 38 days | 6.9 ± 0.5 | 7.1 ± 1.1 | 6.9 ± 0.9 | 6.7 ± 0.4 |
| 57 days | 8.4 ± 1.2 | 8.2 ± 1.1 | 7.9 ± 0.9 | 8.8 ± 0.8 |
| *Age effect: P<0.001* | |  |  |  |
| *Group effect: P=0.820* | |  |  |  |
| *Age * Group effect: P=0.806* | |  |  |  |
| **Stomach weight (tissue + digesta, in grams)** | | | | |
| 18 days | 11.7 ± 3.4 | 10.0 ± 3.6 | 11.5 ± 3.2 | 13.3 ± 1.3 |
| 29 days | 9.0 ± 1.8 | 8.7 ± 1.0 | 9.8 ± 1.6 | 9.6 ± 0.8 |
| 38 days | 7.2 ± 1.4 | 7.0 ± 1.4 | 6.8 ± 1.2 | 6.4 ± 0.7 |
| 57 days | 7.9 ± 1.5 | 7.5 ± 0.9 | 7.7 ± 1.1 | 8.4 ± 1.2 |
| *Age effect: P<0.001* | | |  |  |
| *Group effect: P=0.286* | | |  |  |
| *Age * Group effect: P=0.318* | | |  |  |
| **Small intestine + colon weights (tissue + digesta, in grams)** | | | | |
| 18 days | 14.7 ± 2.2 | 15.3 ± 3.3 | 16.5 ± 2.0 | 15.4 ± 1.6 |
| 29 days | 52.8 ± 8.6 | 52.2 ± 7.0 | 53.6 ± 5.9 | 50.1 ± 7.1 |
| 38 days | 98.6 ± 11.2 | 99.4 ± 15.3 | 99.0 ± 18.6 | 96.2 ± 10.8 |
| 57 days | 170.1 ± 21.2 | 164.8 ± 17.7 | 162.1 ± 27.9 | 170.9 ± 17.3 |
| *Age effect: P<0.001* | | |  |  |
| *Group effect: P=0.921* | | |  |  |
| *Age * Group effect: P=0.741* | | |  |  |
